# Prognostication after cardiac arrest: how EEG and evoked potentials may improve the challenge

**DOI:** 10.1186/s13613-022-01083-9

**Published:** 2022-12-08

**Authors:** Sarah Benghanem, Estelle Pruvost-Robieux, Eléonore Bouchereau, Martine Gavaret, Alain Cariou

**Affiliations:** 1 Medical ICU, Cochin Hospital, Assistance Publique – Hôpitaux de Paris (AP-HP), 27 Rue du Faubourg Saint-Jacques, 75014 Paris, France; 2 Medical School, University Paris Cité, Paris, France; 3 Department of Neurocritical Care, G.H.U Paris Psychiatry and Neurosciences, 1, Rue Cabanis, 75014 Paris, France; 4 Neurophysiology and Epileptology Department, GHU Psychiatry and Neurosciences, Sainte Anne, 75014 Paris, France; 5 After ROSC Network, Paris, France; 6 Paris-Cardiovascular-Research-Center (Sudden-Death-Expertise-Center), INSERM U970, Paris, France; 7 UMR 1266, Institut de Psychiatrie et, INSERM FHU NeuroVascNeurosciences de Paris-IPNP, 75014 Paris, France

**Keywords:** Cardiac arrest, Coma, Disorder of consciousness, Electroencephalogram EEG, Evoked potentials EP, Neuroprognostication

## Abstract

About 80% of patients resuscitated from CA are comatose at ICU admission and nearly 50% of survivors are still unawake at 72 h. Predicting neurological outcome of these patients is important to provide correct information to patient’s relatives, avoid disproportionate care in patients with irreversible hypoxic–ischemic brain injury (HIBI) and inappropriate withdrawal of care in patients with a possible favorable neurological recovery. ERC/ESICM 2021 algorithm allows a classification as “poor outcome likely” in 32%, the outcome remaining “indeterminate” in 68%. The crucial question is to know how we could improve the assessment of both unfavorable but also favorable outcome prediction. Neurophysiological tests, i.e., electroencephalography (EEG) and evoked-potentials (EPs) are a non-invasive bedside investigations. The EEG is the record of brain electrical fields, characterized by a high temporal resolution but a low spatial resolution. EEG is largely available, and represented the most widely tool use in recent survey examining current neuro-prognostication practices. The severity of HIBI is correlated with the predominant frequency and background continuity of EEG leading to “highly malignant” patterns as suppression or burst suppression in the most severe HIBI. EPs differ from EEG signals as they are stimulus induced and represent the summated activities of large populations of neurons firing in synchrony, requiring the average of numerous stimulations. Different EPs (i.e., somato sensory EPs (SSEPs), brainstem auditory EPs (BAEPs), middle latency auditory EPs (MLAEPs) and long latency event-related potentials (ERPs) with mismatch negativity (MMN) and P300 responses) can be assessed in ICU, with different brain generators and prognostic values. In the present review, we summarize EEG and EPs signal generators, recording modalities, interpretation and prognostic values of these different neurophysiological tools. Finally, we assess the perspective for futures neurophysiological investigations, aiming to reduce prognostic uncertainty in comatose and disorders of consciousness (DoC) patients after CA.

## Introduction

The vast majority of patients resuscitated from cardiac arrest (CA) will unfortunately not survive beyond the first days and weeks. Cerebral lesions caused by circulatory interruption and subsequent reperfusion are the main cause of these early deaths [1]. These early deaths remain mainly due to withdrawal-of-life-sustaining-treatments (WLST), presumably secondary to an irreversible hypoxic ischemic brain injury (HIBI). Most often, the severity of these lesions can only be accurately assessed after an observation phase allowing prognostic investigations to be carried out. Among the tools available to clinicians, neurophysiological investigations already occupy a major place. Neurophysiology will probably become even more important in the next future given the progress underway in different directions. In the present review, we aim to present an overview of established data and recent advances coming from neurophysiology in the particular setting of post-cardiac arrest phase.

## Pathophysiology of hypoxic ischemic brain injury

Cerebral damages caused by cardiac arrest are complex and polymorphic, whose the HIBI constitutes the pathophysiological process. Experimental models as well as clinical observation show that injuries leading to HIBI are initiated by circulatory interruption (sudden anoxo-ischemia), but that these primary damages worsen during the first hours (ischemia–reperfusion), thus offering a time-window for therapeutic interventions aiming to limit these phenomena. The mechanisms that lead to the creation of the initial lesions combine to varying degrees dysfunction of the cell membranes, local and systemic inflammation, increase in local excitotoxicity, microvascular abnormalities (associating damage to the endothelium and alteration of vaso-reactivity), alteration of the blood–brain barrier and edematous reactions. The loss of cerebral homeostasis aggravates these lesions, which can be worsened due to various aggressions of systemic origin (abnormalities in blood pressure, arterial oxygen and carbon dioxide content, fever, electrolytic or glycemic disorders). Importantly, some brain regions have an increased susceptibility to these different phenomena. In the extreme, all structures can be potentially affected, explaining the very broad spectrum of clinical manifestations described in patients with HIBI, ranging from transient and totally reversible loss of consciousness to unreactive coma, and even brain death [2, 3].

## Disorders of consciousness after cardiac arrest

Most patients with favorable neurological outcome after CA begin recovering consciousness a few hours after cessation of sedation, awakening being usually defined as a reproductible response to verbal command using the Glasgow Coma Scale (GCS) with a motor score of 6 [2, 4]. Despite this, 80% of patients resuscitated from CA are comatose at ICU admission and nearly 50% of survivors are still unawake at 72 h [2, 3]. Delayed awakening is not rare after sedation weaning and in some situations, awakening may be observed up to 12–25 days after ROSC [5]. Recognized risk factors for delayed awakening are post-resuscitation shock, renal insufficiency, older age, and use of long (i.e., midazolam) vs. short-acting (i.e., propofol) sedative agents [6–8]. Neurological state of consciousness and awareness after CA is highly heterogeneous and subject to time variations [9]. A complete physical examination using adapted scales and rigorous definitions is recommended (see Table 1), first to assess the neurological state as accurately as possible and second to predict consciousness recovery [9, 10]. Coma is defined as a state of unresponsiveness, in which the patient lies with eyes closed and cannot be aroused to respond appropriately to vigorous stimulation [11]. Secondarily, patients may regain arousal (eyes opened) without awareness (pragmatically defined as no reproducible response to command), defining disorders of consciousness (DoC). These DoC entities include vegetative state (VS, also called unresponsive wakefulness syndrome (UWS)) and minimally conscious state (MCS, also called cortically mediated state (CMS))**.** Both VS and MCS are mainly related to a preservation of brainstem arousal functions with persistent impairment of supratentorial networks implicated in consciousness [9, 10]. The distinction between these different patterns of DoC is a key point, as MCS patients are more prone to evolve toward consciousness recovery than VS patients [12–14]. Other DoC and behavioral impairments (as cognitive-motor dissociation (CMD) and delirium) are described in Table 1. Predicting neurological outcome of these patients is important to provide correct information to the patient’s relatives, to avoid disproportionate care in patients with irreversible HIBI, and to avoid inappropriate withdrawal of care in patients with a possible favorable neurological recovery.

**Table 1:** Definitions and scales for disorders of consciousness assessment by the intensivist

Behavioral criteria	Definitions and pragmatic criteria for diagnosis	Scales and/or scores
Coma [11]	**No wakefulness/arousal (no spontaneous eye opening)** **No awareness of self or environment**	Glasgow coma scale [17] Four score or RASS in mechanical ventilated patients [155, 156]
VS also known as UWS [9, 17, 157]	**Wakefulness/arousal preserved (spontaneous eye opening)** **No awareness of self or environment** No sustained, reproductible, purposeful behavioral responses to external stimuli No language comprehension or expression **Could presented reflex behavioral signs as sound localization** Relatively preserved hypothalamic/brainstem autonomic functions Variably preserved cranial-nerve and spinal reflexes	CRS-r [158]
MCS also called CMS [157, 159]	**Wakefulness/arousal preserved (spontaneous eye opening)** **Fluctuating awareness with reproductible, purposeful behavioral responses to external stimuli as visual pursuit, reaching for objects, contingent behavior or orientation to noxious stimulation** Does not necessary correspond to “residual consciousness” but at least demonstrates contribution of cortical networks in the behavioral responses (CMS)	CRS-r [158]
Emergence from MCS (E-MCS) [9, 17, 157]	**Wakefulness/arousal preserved (spontaneous eye opening)** **Sign of awareness: following commands, intelligible verbalization or intentional communication**	CRS-r [158]
Cognitive-motor dissociation [93]	**Wakefulness/arousal preserved (spontaneous eye opening)** No or very limited behavioral evidence of awareness but empirical evidence of command-following via fMRI, qEEG or similar indirect measurements of brain response to spoken language	Dissociation between behavioral motor dysfunction and the preserved higher cognitive functions only measurable by functional techniques [89, 92, 92, 160]
Delirium [161]	**Acute and fluctuating disturbance of consciousness**: attention and impairment of cognition associated with motor hyperactivity or hypoactivity	CAM–ICU and/or ICD-SC [161–163]

Pragmatic criteria of DoC assessment for the intensivist are shown in bold.

*CAM-ICU* Confusion Assessment Method in the ICU, *CMS* Cortically mediated state, *CRS-r* Coma Recovery Scale-revised, *DoC* disorders of consciousness, *E-MCS* emergence from minimally conscious state, *fMRI* functional magnetic resonance imaging, *ICD-SC* Intensive Care Delirium Screening Checklist, *MCS* minimally conscious state, *qEEG* quantitative EEG, *RASS* Richmond Agitation-Sedation Scale, *UWS* unresponsive wakefulness syndrome, *VS* vegetative state

## The challenge of neuro-prognostication

To date, most of the studies exploring indicators of prognosis after CA have focused on unfavorable outcome (UFO) prediction. The challenge is to identify markers with the highest specificity and the lowest false positive rate (FPR), to minimize the possibility of wrong prediction [4]. However, these studies presented several risks of bias. First, the lack of blinding could induce self-fulfilling prophecy, as test results are used for decisions of WLST. To limit this risk, the current guidelines are based on the most robust indicators, which are also combined with each other (i.e., never used in isolation). Another potential bias is the use of different scales to assess neurological outcome according to studies. The most employed scale is the Cerebral Performance Categories (CPC). The CPC is directly adapted from the Glasgow Outcome Scale (GOS), although the GOS only considers disabilities related to brain injury. The CPC ranges from CPC 1 (no or minimal disability) to CPC 5 (death), CPC 1–2 being usually considered as favorable outcome [15]. Despite its widespread use, the CPC scale has a limited accuracy for discrimination of mild and moderate disabilities and does not assess psycho-cognitive functions. The CPC also does not discriminate neurological from non-neurological causes of death, although a large multicenter study suggested that in-ICU death after awakening is observed in 4.2% of CA patients who are misclassified in the CPC 5 level. The use of the «best CPC» observed during the hospital stay (and not the CPC at discharge) could further limit this bias [16]. In several recent studies, more accurate scales are used, such as the Glasgow Outcome Scale-Extended (GOS-E, ranging from 1 to 8) or the modified Rankin scale (mRS, from 0 to 6) [17, 18]. Third, the timing of neurological assessment is another major bias as it differs widely among studies, although neurological outcome may further improve over time. In a large prospective study examining ICU survivors, the CPC level at hospital discharge improved in 14.5% of subjects at 6 months, mainly due to patients who were initially CPC 3 and who then evolved to CPC 2 [19], highlighting that the neurological function should not be assessed too early [20]. Finally, an important source of bias for prognostication is the remaining effect of sedatives and analgesia drugs. Sedation is mainly used to permit post-resuscitation care (mechanical ventilation, invasive procedures, temperature management) but it may alter prognostication in different ways [21, 22]. Sedation may delay awakening [2, 6], confound clinical examination (i.e., pupillary and corneal reflexes, that are both robust markers of poor outcome) and alter some neurophysiological markers (Table 2) [23]. These different points encourage the use of light-to-moderate dose of sedation with short-acting drugs (i.e., propofol) [24]. To minimize the risk of a falsely pessimistic prediction, recent guidelines recommend a multimodal approach for prediction of UFO, using at least two markers among: loss of pupillary and corneal reflexes, clinical status myoclonus, highly malignant electroencephalogram (EEG), bilateral abolition of N20 on somato-sensory evoked potential (SSEP), high release of biomarkers (neuron-specific-enolase (NSE) > 60 µg/L at 48 or 72 h) and identification of specific damages using brain imaging [4]. However, a recent study highlighted that this algorithm allows a classification as “poor outcome likely” in 32%, the outcome remaining “indeterminate” in 68% [25].

**Table 2:** Light-to-moderate sedation (< 3 mg/kg/h of propofol or midazolam equivalent) effect and recommended timing of EEG and EPs assessments [9, 30, 31, 46]

	Light-to-moderate sedation effect on EEG and EPs	Recommended timing of EEG and EPs assessment
EEG	Decrease frequency: slow background Decrease amplitude: low voltage Fast rhythms (mainly with benzodiazepines)	Performed at 72 h after CA Could be performed earlier, at 24 h after CA (if possible, without sedation) Use cEEG monitoring if available in ICU
SSEP	N20: poorly affected by sedation Decrease SSEP N20–P25 amplitudes	Performed at 72 h after CA Could be performed under sedation if needed (do not use amplitudes results)
BAEP	BAEP: no influence of sedation BAEP latencies: increased by sedation	Performed at 72 h after CA Could be performed under sedation if needed
MMN	Risk of false negative if performed under sedation Need sedative drugs elimination	Performed at 72 h after CA Performed 48 h after sedation weaning
P300	Risk of false negative if performed under sedation Need sedative drugs elimination	Performed at 72 h after CA Performed 48 h after sedation weaning

*BAEP* brainstem auditory evoked potentials; *EEG* electroencephalogram; *ERP* event-related potentials; *MMN* mismatch negativity; *SSEP* somato-sensory evoked potential. *ICU* intensive care unit

The crucial question is now to know how we could improve the assessment of both poor but also favorable outcome prediction [26]. This review proposes an overview of neurophysiological markers potentially interesting to reduce prognostic uncertainty in comatose and DoC patients after CA.

## Methods

Regarding prognostic value assessment, we considered for inclusion only clinical studies, published as full text articles between 1995 and 2022, filtered by “English language” and “humans”. We excluded case reports, commentaries, publications with less than 10 patients, abstracts, editorials, or letters. We included studies on adult patients (> 18 years old) presenting a comatose state or a DoC after CA. Search strategy included MEDLINE via Pubmed database. We assessed separately the following neurophysiological indicators: “EEG”, “SSEP”, “brainstem auditory evoked potential (BAEP)”, “middle latency auditory evoked potential (MLAEP)”, “event-related potential (ERP)” or “cognitive evoked potential”, “P300”, “P3” and “mismatch negativity (MMN)”.

## Electroencephalography

### EEG signal generator

The EEG is the record of brain electrical fields. It is characterized by a high temporal resolution but a low spatial resolution. EEG signals are mainly explained by the postsynaptic activities (excitatory and inhibitory) of synchronously activated neurons that generate open electrical fields within the extracellular space [27, 28]. To reach the scalp, brain electrical signals must pass several layers of tissues with different electrical properties. This implies that what is recorded using surface EEG is an attenuated and transformed image of brain sources. The spatial EEG sampling is represented by the number of surface electrodes, while the temporal sampling corresponds to the sampling rate (usually around 256 Hz). It is possible to enhance the number of surface electrodes (up to 256), the sampling rate (up to 2000–3000 Hz) to perform high resolution EEG [29, 30].

EEG is sensitive for both radial and tangential components. Neuronal assemblies that are functionally interconnected constitute a functional brain workspace. In general, the neurons that constitute those assemblies are interconnected by feedforward and feedback loops. Traditionally, EEG signals are described in terms of frequency bands: infra-slow (< 0.2 Hz), *δ* (0.2–3.5 Hz), *θ* (4–7.5 Hz), *α* (8–13 Hz), *β* (14–30 Hz), *γ* (30–90 Hz) and high-frequency oscillations (> 90 Hz). In normal brain are thus observed an alpha rhythm (between thalami basal ganglia and posterior cortex areas), a mu rhythm (between thalamic and central areas), and spindles during slow waves sleep (between thalami and large cortical areas). A predominant posterior alpha rhythm is usually observed in awake and resting normal condition [28, 31].

**Fig. 1:**
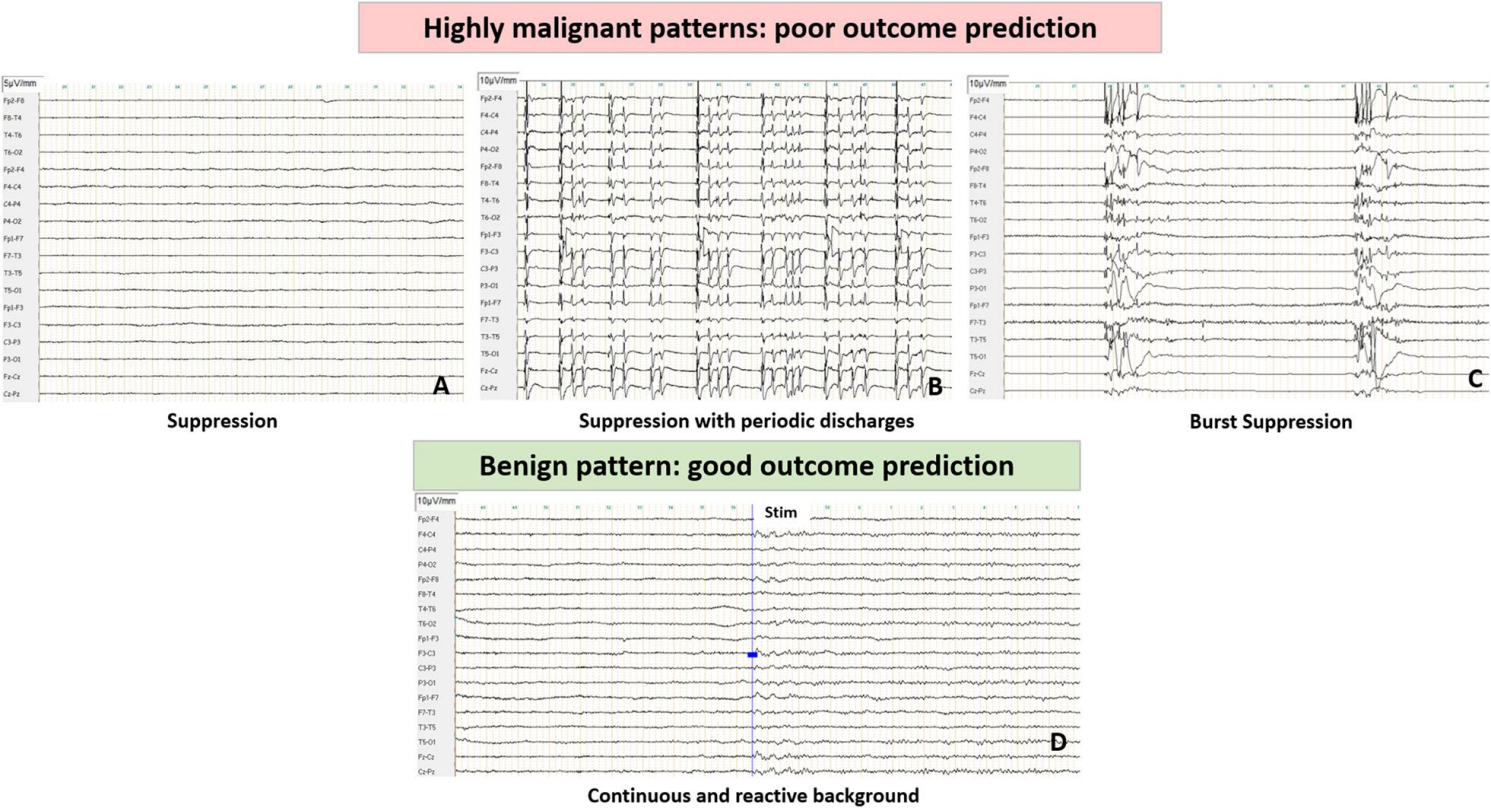
Highly malignant and benign EEG patterns (adapted from Westhall et al. [58] and ACNS terminology [32]). **A**, **B**, **C** Figures represented EEG longitudinal montage showing highly malignant patterns. **A** Suppression, defined as suppressed EEG background (amplitude  < 10 µV all the time of the recording) without discharges. **B** Suppression with periodic discharges, defined as a suppressed EEG background with continuous periodic discharges (spikes, poly-spikes, sharp or waves). **C** Burst suppression, defined as suppression periods (< 10 µv) constituting  > 50% of the recording with “burst”. **D** EEG longitudinal montage showing benign EEG, defined as the absence of a malignant or highly malignant features namely continuous or nearly continuous and reactive EEG background. Blue line indicated the nociceptive stimulus (nail pressure), inducing amplitude and frequency modifications and defining reactivity when reproductible

In comatose patients after CA, EEG background may display a large spectrum of abnormalities. The severity of HIBI is correlated with the predominant frequency but also with background continuity. For example, burst suppression is defined as a pattern of suppression background (during 50–99% of the record) that alternates with higher voltage activities (called “burst”, with two sub-types according to the identical or non-identical bursts characteristics) (Fig. 1) [32]. Source analysis and animal models support the theory that a deafferentation between cortical and subcortical structures exists in suppression period compared to burst phases. Indeed, brainstem “arousal system” (i.e., ascending reticular activating system) projections toward the thalamus and then cortical areas provide a key coupling for arousal, awareness and cognitive processing [33]. The central thalamus (intra laminar and para laminar nuclei) also receives upward projections from the brainstem that control the activity of many cortical and thalamic neurons. These projections are present only during the burst phases, whereas there is no coherence or interaction between cortical and subcortical structures during suppression periods [34, 35]. In DoC patients after CA, visual EEG analysis is usually not sufficient to discriminate between VS and MCS [9, 36]. Despite this, recent studies suggest that all stages of sleep (with reliable neurophysiological features, such as periods of consolidated slow wave sleep spindles and rapid eye movements) are only observed in MCS. These results suggest that sleep spindles reflect a relative preservation of thalamo-cortical connectivity, although the prognostic value of these patterns for recovery of consciousness prediction remains unknown [9, 37].

### EEG recording modalities

Recent surveys examining current neuro-prognostication practices after CA reveal that EEG is the most widely used tool for assessing the severity of HIBI, both in Europe (63%) and United States (94.4%) [38, 39]. The timing of EEG recording remains largely heterogeneous among studies, ranging from 12 h (i.e., under sedation) to 7 days after CA [26, 40, 41]. As the EEG reflects the “real time” cortical activity, the pattern evolution over time could be an interesting prognostic information, particularly for EEG patterns predictive of UFO which may disappear over time [42–45]. Moreover, the prognostic value of EEG patterns could differ according to the timing of assessment and recent studies suggest that prognostic value of early EEG (obtained 12 to 24 h after CA) may be better than later recordings (see below). These results suggest that physicians should carry out EEG recording at 24 h and at 48–72 h after CA [31]. Importantly, neuro-prognostication issue should only be addressed in unresponsive state patients 72 h after CA and 48 h after sedation weaning. It suggests that early EEG recorded at 24 h after CA could be secondarily integrated into this neuro-prognostication process [4].

Routine discontinuous video-EEG recording is performed using 21 electrodes (minimum of 9 electrodes) including central–medial (Cz) electrode, during 20 min and coupled with a video recording [31, 46]. Recent data encourage the use of continuous EEG (cEEG) or reduced montages EEG devices [30, 47]. Whether cEEG is superior to routine intermittent EEG remains debated. In two large prospective studies, cEEG does not confer any advantage over two intermittent EEG regarding neurological outcome or time to death [48–51]. Despite this, international guidelines suggest that cEEG should be performed if available for seizure detection, treatment monitoring and prognostic assessment of HIBI [9, 30, 31]. Otherwise, the use of a limited-channel cEEG monitoring (4 or 6 frontal electrodes) could detect the most common EEG patterns associated with poor and good outcome [52, 53], although eye movement artifacts over bi-frontal electrodes could induce false positive detection of periodic discharges [52].

### EEG interpretation and classification

**Fig. 2:**
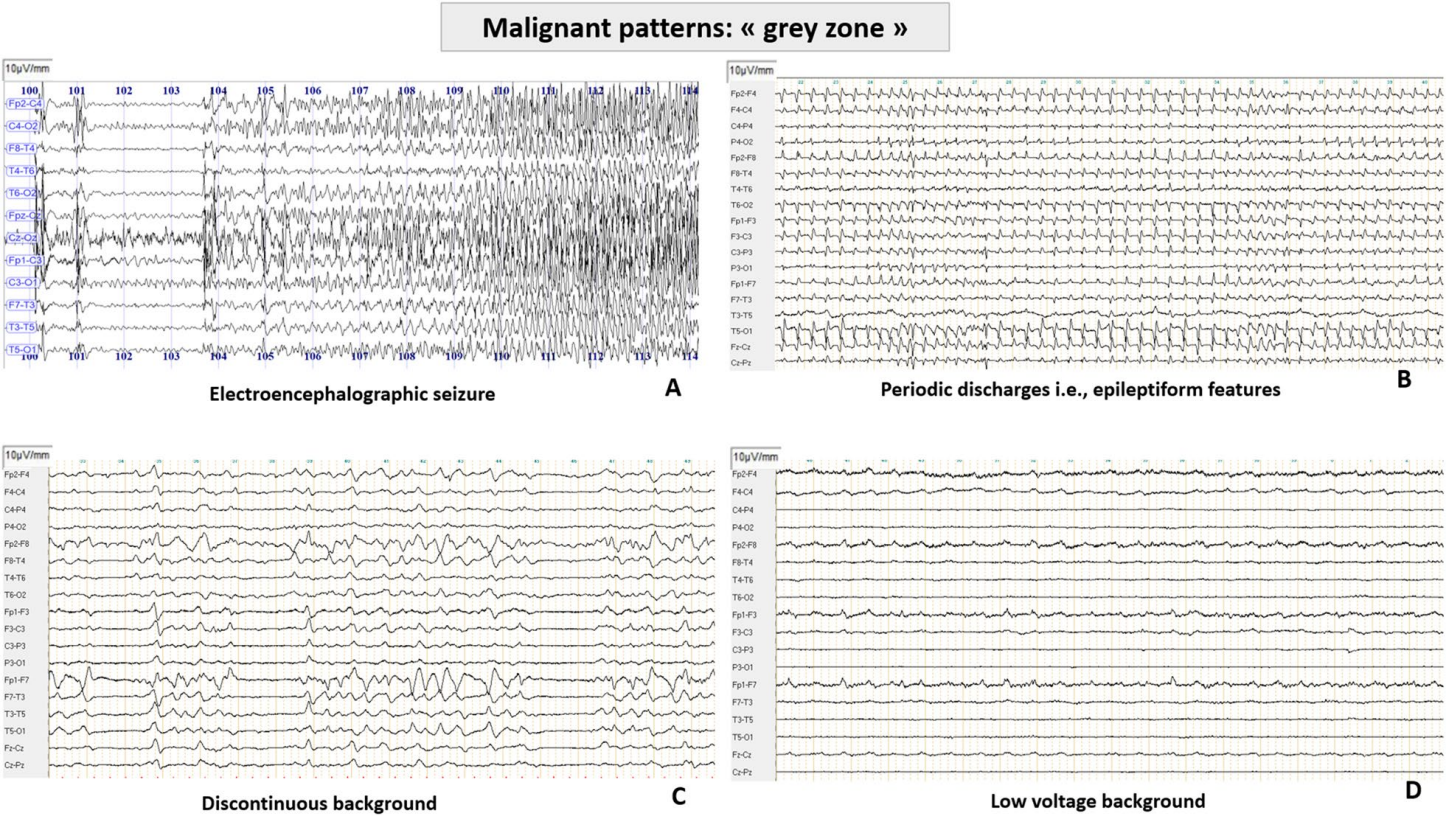
EEG longitudinal montages showing typical malignant patterns (adapted from Westhall et al. [58] and ACNS terminology [32]). **A** Electroencephalographic seizure with high frequency (> 2.5 Hz) generalized spikes, more broadly defined as pattern with epileptiform discharges  > 2.5 Hz for  ≥ 10 s or any pattern with definite spatial and temporal evolution lasting  ≥ 10 s. **B** Generalized periodic pattern (i.e., epileptiform features) defined as non-evolving and low frequency (<2.5 Hz) periodic discharges (spike, sharp or wave)  during > 50% of the recording, without suppressed background. **C** Discontinuous background defined as suppression periods (< 10 µV) constituting  > 10% but  < 49% of the recording. **D** Low voltage background defined as amplitude of the background  < 20 µV. Unreactive EEG is not illustrated here

Analysis of EEG is a complex task, requiring standardization in interpretation and classification. Concerning interpretation, EEG findings are typically categorized according to four determinants: background, sporadic epileptiform features (also called rhythmic or periodic patterns (RPPs)), electroencephalographic seizures and reactivity (Figs. 1, 2). Regarding background activity, the severity of HIBI is correlated with the predominant frequency, amplitude and continuity, ranging from slow background (delta or theta frequency), low voltage (amplitude  < 20 µV), discontinuous (amplitude  < 10 µV during 10–49% of the EEG recording), burst suppression (< 10 µV during  > 50% and  < 99%, with identical or non-identical bursts) or suppression background (< 10 µV all the time). Sporadic epileptiform discharges, electroencephalographic seizures and unreactive EEG could also reflect HIBI, with different degrees of severity.

Concerning classification, Young and Synek classifications were historically proposed for all critically ill patients [54, 55]. More recently, the American Clinical Neurophysiology Society (ACNS) critical care EEG terminology [32] was adapted by Hofmeijer et al. [56], Gaspard et al. [57] and Westhall et al. [58], resulting in three main categories: highly malignant, malignant and benign EEG [58], 56 (Figs. 1, 2) (Table 3). This ACNS terminology is now the most employed classification for prognostication after CA [1, 26, 45], although it does not describe pathophysiological mechanisms of EEG abnomalities [59, 60]. Nevertheless, using this classification could help to standardize interpretation, and may facilitate collaboration between intensivists and neurophysiologists.

**Table 3:** Definitions and prognostic value of each EEG patterns, adapted from ACNS terminology and Westhall et al. classification [32, 58]

EEG	Patterns	Definition	False positive rate (%) CI95(%) for poor outcome prediction (CPC 3–4–5)
**Highly malignant ≥ 24 h after CA**			
	Suppression	Suppressed background (< 10 µV) during all the recording No discharge	0% CI95(0–11.7) [41, 42, 58, 61–63, 65, 123]
	Suppressed background + periodic discharges	Suppressed background (< 10 µV) during all the recording, with periodic discharges	0% CI95(0–23.1) [41, 42, 58, 63]
	Burst suppression	Alternance of: - Suppressed background (suppression period > 50% and < 99% of the recording) with identical burst Or alternance of: - Suppressed background (suppression period > 50% and < 99% of the recording) with non-identical burst	Suppressed with identical burst: 0% CI95(0–20.6) [41, 42, 58, 61–63, 65, 123] Suppressed with non-identical burst: 0–1.4% CI95(0–3.1) [42]
**Malignant ≥ 12 h after CA**			**False positive rate (%) CI95(%) for poor outcome prediction (CPC 3–4–5)**
Malignant periodic or rhythmic patterns	Periodic discharges Also called epileptiform features	Abundant non-evolving* and low frequency (0.5–2.5 Hz) periodic discharges (spike, sharp or wave) during > 50% of the recording No suppressed background	Early (< 24 h after CA): 0–3% CI95(0–34.8) [62, 65, 67–69, 164, 165] Late (≥ 24 h after CA): 0–33.3% CI95(0–70.1) [42–44, 61, 166]
	Rhythmic discharges Also called epileptiform features	Abundant non-evolving* rhythmic discharges during > 50% of the recording No suppressed background	Early (< 24 h after CA): 0–3% CI95(0–34.8) [62, 65, 67–69, 164, 165] Late (≥ 24 h after CA): 0–33.3% CI95(0–70.1) [42–44, 61, 166]
	Unequivocal electrographic seizure or electrographic status epilepticus	Epileptiform discharges > 2.5 Hz for ≥ 10 s or Any pattern with definite temporo-spatial evolution and lasting ≥ 10 s Electrographic seizures for ≥ 10 continuous minutes or for a total duration of ≥ 20% of any 60-min period of recording	0–17.4% CI95(0–26.7) [41, 43, 50, 71, 74, 167–169]
Malignant backgound	Discontinuous background	Suppression periods (< 10 µV) constituting > 10% but < 49% of the recording	0–13.8% CI95(0.1–48.2) [42, 62, 82][65]
	Low voltage background	Amplitude < 20 µV	0–12.1% CI95(0–29.2) [42, 58, 63, 76, 170]
	Unreactive background	Absence of reproductible modification for both sound, visual and sensory stimuli	0–50% CI95(0–70.9) [41, 58, 61, 67, 71, 76, 84, 86, 166]
**Benign ≥ 12 h after CA**			**Specificity (%) CI95(%)** **for good outcome prediction (CPC1–2)**
	Continuous normal-voltage background or nearly continuous normal-voltage background without discharges and reactive EEG	Continuous background during all the recording Nearly-continuous: Suppression periods (< 10 µV) constituting < 10% of the recording reactive EEG: Change in cerebral EEG activity to stimulation, include change in voltage or frequency (including attenuation of activity)	Continuous or nearly continuous EEG: 56.5–100% CI95(45.3–100) [26, 44, 56, 58, 61, 78] Reactive EEG: 57.1–85% CI95(37.2–93) [26, 42, 44, 78, 86, 105, 171]

^*^non-evolving: no temporal or spatial evolution.

*CI* confidence interval, *EEG* electroencephalogram

### ACNS classification definition and prognostic value

Table 3 displays the ACNS terminology adapted by Westhall et al. and the prognostic value for each pattern [32, 58]:

- **Highly malignant patterns** correspond to three main patterns: suppression, suppression with periodic discharges and burst suppression. These patterns are recognized as robust markers of UFO (i.e., FPR close to 0% confidence interval CI95%(0–22.1), sensitivity 47–97%) [41, 42, 61–65]. Consequently, these patterns are considered as one of the six prognostic markers for poor outcome prediction in the last 2021 ERC/ESICM recommendations [4].
- **Malignant patterns** include five different aspects: 1/Abundant rhythmic or periodic discharges (RPPs) (also called epileptiform features); 2/electroencephalographic seizures or status epilepticus (SE); 3/discontinuous background; 4/low voltage (< 20 µV); 5/unreactive EEG (Fig. 2). Prognostic value of malignant EEG remains very heterogeneous among the different patterns. Most studies assess the prognostic value of these different patterns all together and not their individual performance [41, 66]. Early (< 24 h after CA) non-evolving and low frequency (0.5–2.5 Hz) generalized periodic or rhythmic discharges appear to be the most robust predictors of UFO, with an FPR 0–3% despite a large CI95%(0–34.8) [62, 65, 67, 68]. Importantly, a recent multicentric randomized study highlighted that an aggressive anti-epileptic treatment of these epileptiform features does not improve neurological recovery, as compared with standard of care (CPC3–5 in 90% and 92%, respectively) [43]. To note, a minority of patients with « late» epileptiform patterns (i.e., appearing after sedation weaning ≥ 24 h after CA) may present a favorable outcome if subsequently treated [69, 70], suggesting that early epileptiform features (< 24 h) could be associated with a worse neurological outcome [63]. It is of importance to underline that these “epileptiform patterns” (i.e., periodic or rhythmic discharges) must be well-differentiated from electroencephalographic seizures, as seizures are defined as high frequency (> 2.5 Hz) epileptiform discharges for ≥ 10 s or any pattern with a temporo-spatial evolution of the discharges lasting ≥ 10 s [32]. Electroencephalographic seizures and SE are mainly associated with UFO (FPR 0–17.4%, CI95%(0–26.7)) [43, 63, 68, 71, 72]. Importantly, two studies report a favorable outcome in patients with SE, these patients presenting no other markers of unfavorable outcome [73, 74]. These results suggested that isolated SE is probably not sufficient to accurately predict a poor outcome. Others malignant features and prognostic values are described in Table 3. Ultimately, malignant patterns are associated with UFO but with high FPR and large CIs. Consequently, these markers could only be considered as « minor criteria» of severe HIBI (Fig. 2).
- **Benign patterns** are defined as continuous or nearly continuous and normal voltage background without any discharges. These patterns are predictive of good outcome with a moderate to high specificity (56.5–100%) and a variable sensitivity (29.6–97.9%) among studies [44, 56, 58, 61].

### EEG prognostic value according to sedation, hypothermia and timing of recording

About prognostic value of EEG patterns, a major and already debated point remains the potential confounding effect of sedation. Most sedative drugs have similar effects on EEG spectrum, namely, decreasing frequency and amplitude. More specifically, light-to-moderate dose of benzodiazepines as midazolam lead to diffuse fast rhythms, whereas both high dose of midazolam and propofol generates discontinuous or even burst suppression patterns, which are both an important predictor of UFO [4, 75]. It is of importance to underline that burst suppression is usually observed with higher doses than those generally used for the management of post-CA patients [42, 76, 77]. A large post hoc analysis of a prospective multicentric study highlights that light-to-moderate sedation (i.e., maximum doses of 3.0 to 3.5 mg/kg/h of propofol and 63 to 68 µg/kg/h of midazolam) does not affect the prognostic value of EEG, despite an effect on amplitude reduction, dominant frequency and background continuity. Interestingly, patients who displayed a continuous background were sedated with higher median doses (2.67 mg/kg/h) compared to patients with burst suppression or suppression patterns (2.07 mg/kg/ and 1.94 mg/kg/h, respectively) [42]. Finally, more and more studies suggest that light-to-moderate sedation (i.e., for temperature management) does probably not significantly impair the prognostic accuracy of the early EEG (i.e., 24 h after CA) compared to recordings carried out after 48–72 h [42, 75, 76, 78]. Caution is needed when patients required deep sedation or the use of two different drugs, as the association of midazolam and propofol decreased the probability to detect benign EEG patterns [42]. Regarding temperature effect on EEG, it must be recognized that its own effect remains difficult to assess independently from the effect of sedation, as sedative drugs are almost systematically used during the first 24 h of target temperature management (TTM) after CA. Indeed, EEG could be sensitive to hypothermia, inducing a decrease of the amplitude and frequency of the EEG background (around 33 °C), a burst suppression (between 33 and 31 °C) or an isoelectric pattern (at 22 °C) [79]. These different levels of hypothermia remain lower than those currently recommended for TTM management [24, 80]. Furthermore, temperature management at 36 °C does not lead to significant EEG change [31].

Prognostic value of EEG pattern could also differ according to the timing of EEG assessment [45]. About poor outcome prediction, large prospective studies suggest that a highly malignant pattern 24 h after CA is highly correlated with severe HIBI (FPR = 0%), leading to a reduced length of ICU stay and a small cost reduction [81]. Regarding prediction of good outcome, a benign EEG recorded between 12 and 24 h after CA seems to be strongly associated with favorable outcome, with a higher specificity (between 86% and 95%) [42, 56, 67] compared to a benign EEG observed at 72 h (specificity between 65% and 78%) [42, 67]. Finally, more and more studies suggest that an early EEG recording 24 h after CA might carry a higher prognostic value compared to later recordings (i.e., after 48 h), at least when a highly malignant or benign pattern is observed, even under “light-to-moderate” dose of sedation [42, 82].

### EEG reactivity

EEG reactivity (EEG-r) is defined as a transient reproductible amplitude or frequency change in response to stimulation [32]. Examination of EEG-r requires integrity of peripheral (sensory receptors) to central structures (medullar, brainstem, sub cortical and cortical networks) [33]. Modalities of stimulation usually include auditory, visual and tactile stimuli, although nature and strength protocols may differ [44, 83]. Visual inspection of EEG tracing allows to classify the background as reactive or unreactive to the stimulation. Muscle activities, eye blinks and stimulus-induced rhythmic or periodic discharges (SIRPIDS) are usually not qualified as reactivity [84, 85].

Regarding its prognostic value, loss of EEG-r tends to be associated with UFO, regardless of concomitant sedation [33, 86]. However, the FPR remain heterogeneous across studies (FPR = 0–50% CI95% (0–70.9)) limiting the use of this criteria as a robust marker of UFO. Otherwise, presence of EEG-r is associated with favorable outcome, with a specificity between 57.1% and 85% and a sensitivity between 40% and 91% [42, 44, 67, 78, 86]. Two large prospective studies also suggest that timing of assessment is of importance, as an early (12 h after CA) reactive background seems to be more specific of good outcome as compared to late assessment (48–72 h after CA) (predictive positive value PPV = 88.9% vs 55.6%, respectively [78]). At that time, the use of EEG-r is limited in different ways. First, the protocol of stimulation for EEG-r recording (stimulus type, duration and number of stimulation) is not standardized [87, 88]. Second, EEG-r interpretation is subject to inter-rater variability [44]. Admiraal et al. propose a new definition of EEG-r as any of five stimuli (sternal rub, clapping, calling patient’ name, nasal tickle and eye opening) induced a change in EEG at least twice out of the three stimuli applications [44, 83]. All together, these different limitations suggest that an unreactive background is associated with UFO but should not be used alone, as it is a “minor criteria” of severe HIBI [41].

### EEG quantitative analyses

Considering that visual analysis remains subjective, quantitative analyses of the EEG signals (qEEG) using machine learning have been recently developed [89]. These qEEG modalities may be broadly categorized into spectral and connectivity analyses [90].

Spectral analyses are based on the partition of the EEG signal into different frequency bands of interest using fast Fourier transformation. The power spectral density, which corresponds to the relative distribution of the different frequency bands, is used to characterize the EEG spectrum over time. This spectral analysis density allows an “automatic” detection of high frequency picks over long time periods of continuous monitoring. As these high frequency picks may reveal seizure or status epilepticus, spectral analysis could be an interesting tool to assess outcome. Regarding prognostic value, low-power spectral values in alpha band (around 10 Hz) recorded on an early EEG seems to be highly specific of UFO (sp = 100%) [91]. These low power spectral values possibly reflect impairment of cortico-thalamic connections. Recent studies also suggested that power spectral density analyses could help to assess CMD patients, during an active EEG paradigm. This “motor command protocol” compared the EEG responses during (“*keep opening and closing your hand*”) and after (“*stop opening and closing your hand*”) motor commands, EEG signal (in selected frequency ranges) being significantly different between the two commands in CMD patients [89, 92, 93].

Connectivity analyses can be divided into functional and effective connectivity. Functional connectivity refers to a statistical dependence between two signals, which can be assessed by different linear or non-linear measures (i.e., correlation, coherence, phase, power, mutual information), while effective connectivity refers to the causal influence of one neural network over another [94–97]. However, effective connectivity is much more difficult to ascertain and metrics such as Granger causality or transfer entropy can be used with many limitations [94]. EEG connectivity could also be influenced by concomitant sedation. For example, propofol induces a reorganization of neural networks with an increased connectivity in the delta band in posterior regions [99, 100] and a persistent synchronous alpha activity in anterior regions, which is a sign of changes in the dynamic of thalamo-cortical loops [101, 102].

These qEEG analyses have also been integrated into composite prognostic markers combining spectral (i.e., frequency) and connectivity (i.e., entropy) analyses, such as the revised cerebral recovery index (rCRI). Such combination extracted from resting-state EEG has been reported to predict UFO (sp = 100%, se = 66%) [103][98]. Furthermore, automated machine learning algorithms of candidate qEEG reactivity markers have recently shown a higher predictive value for poor and good outcome compared to visual analyses [104, 105]. Despite these different results, qEEG analyses are not widely employed in routine practice, notably because of a lack of availability and also because validation studies in larger cohorts are required.

## Evoked potentials

### EP signal description

**Fig. 3:**
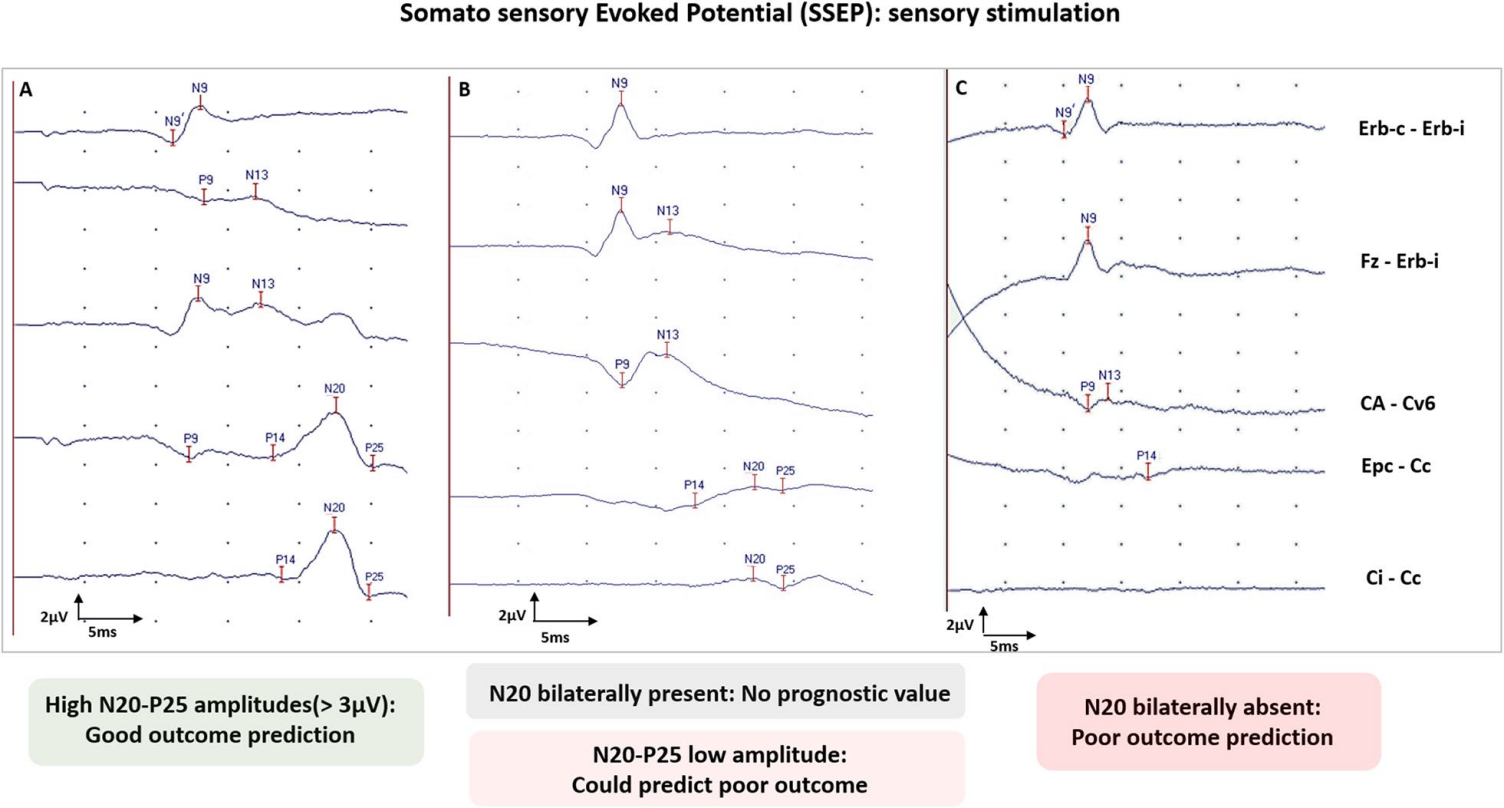
Somato-sensory evoked potential (SSEP) interpretation and prognostic value in comatose and DoC patients after CA. SSEPs five channels recording with N9 (proximal part of the median nerve) N13 (posterior columns of the spinal cord) P14 (cervico-medullar level), N18 (subcortical), N20 and P25 (primary somatosensory cortex). *Erb-i* Erb point ipsilateral, *Erb-c* Erb point contralatera, *Fz* Midline frontal electrod, *CA-Cv6* cervical anterior and cervical posterior C6 electrode, *Epc-Cc* centro-parietal electrode contralateral to the stimulation (C’3 or C’4) and the shoulder contralateral to the stimulatio, *C’3–C’4* centro-parietal electrode ipsilateral and contralateral to the stimulation. Only one side is here presented. Pre-requisite for ERP interpretation: N9 and N13 should be present. Use of neuromuscular blockage agents is recommended if artefacts limit the recording. Part **A** Shows that N20 and P25 are present with a high N20–P25 amplitude. Part **B** Shows that N20 and P25 are present with a low N20–P25 amplitude. Part **C** Shows an absence of N20 and P25, with preserved N9 and N13 responses

**Fig. 4:**
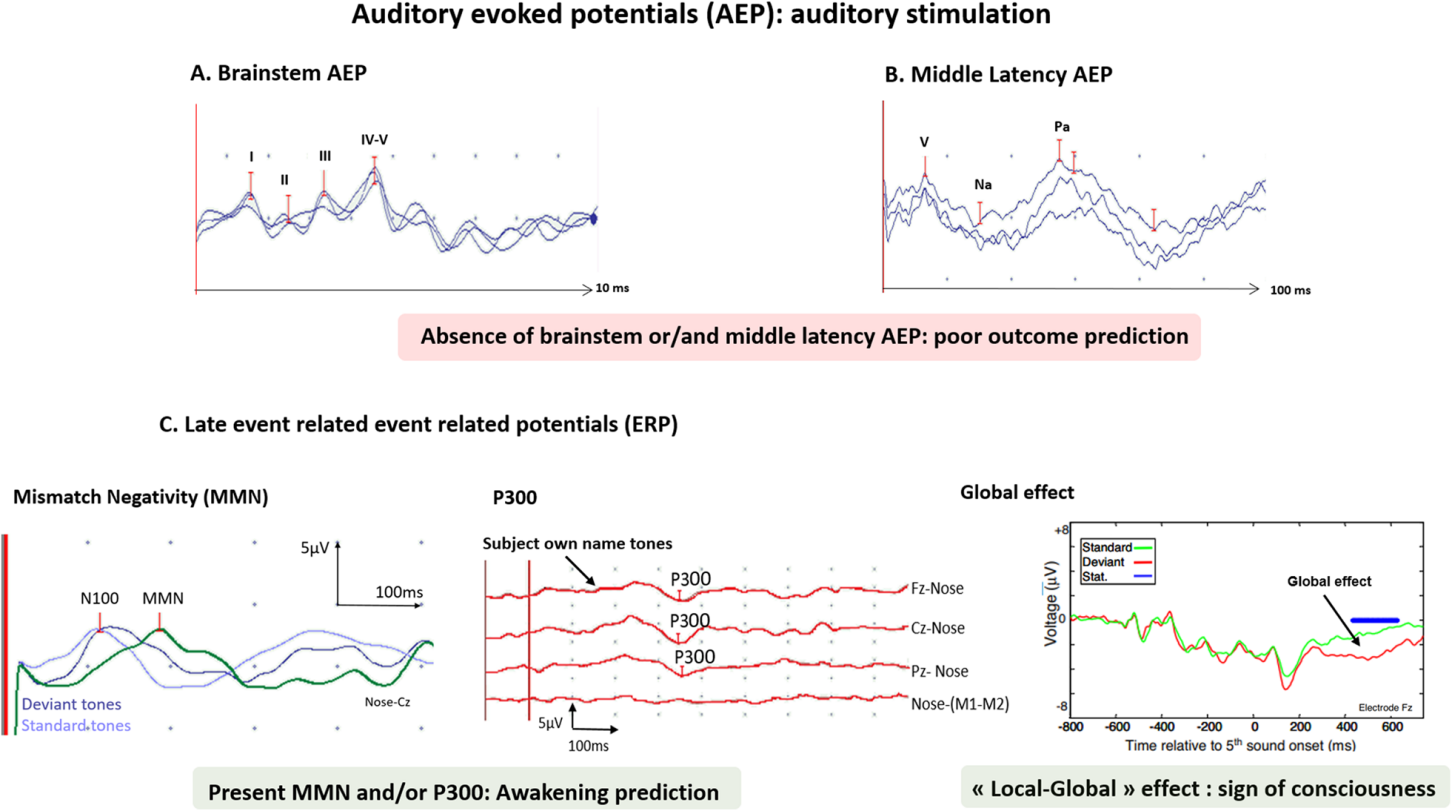
Auditory evoked potentials interpretation and prognostic value in comatose and DoC patients after CA. Pre-requisite for ERP interpretation: presence of BAEP, MLAEP and N100 (primary auditory cortices responses). Use of neuromuscular blockage agents is recommended if artefacts limit the recording. Part **A** Shows brainstem auditory evoked potentials (BAEP) (auditory nerves and brainstem integration of the auditory stimuli). Part **B** Shows middle latency auditory evoked potential (MLAEP). Part **C** Shows late auditory ERPs with Mismatch Negativity (MMN), P300 and “local–global” effect. MMN is elicited by an auditory passive “oddball” paradigm (series of standard frequent tones and deviant infrequent tones). MMN is obtained by subtracting the ERP of the deviant and standard tones. P300 is elicited by the same “oddball” paradigm with intermix of scarce subject’s own-name stimuli. «Local–global» effect (figure adapted from Bekinschtein et al. PNAS, 2009 [140]) is recorded during an active counting task (tones with local and global deviations), patients having to count these “global deviations” which elicit a spatially cerebral distributed response called the “global effect”

**Fig. 5:**
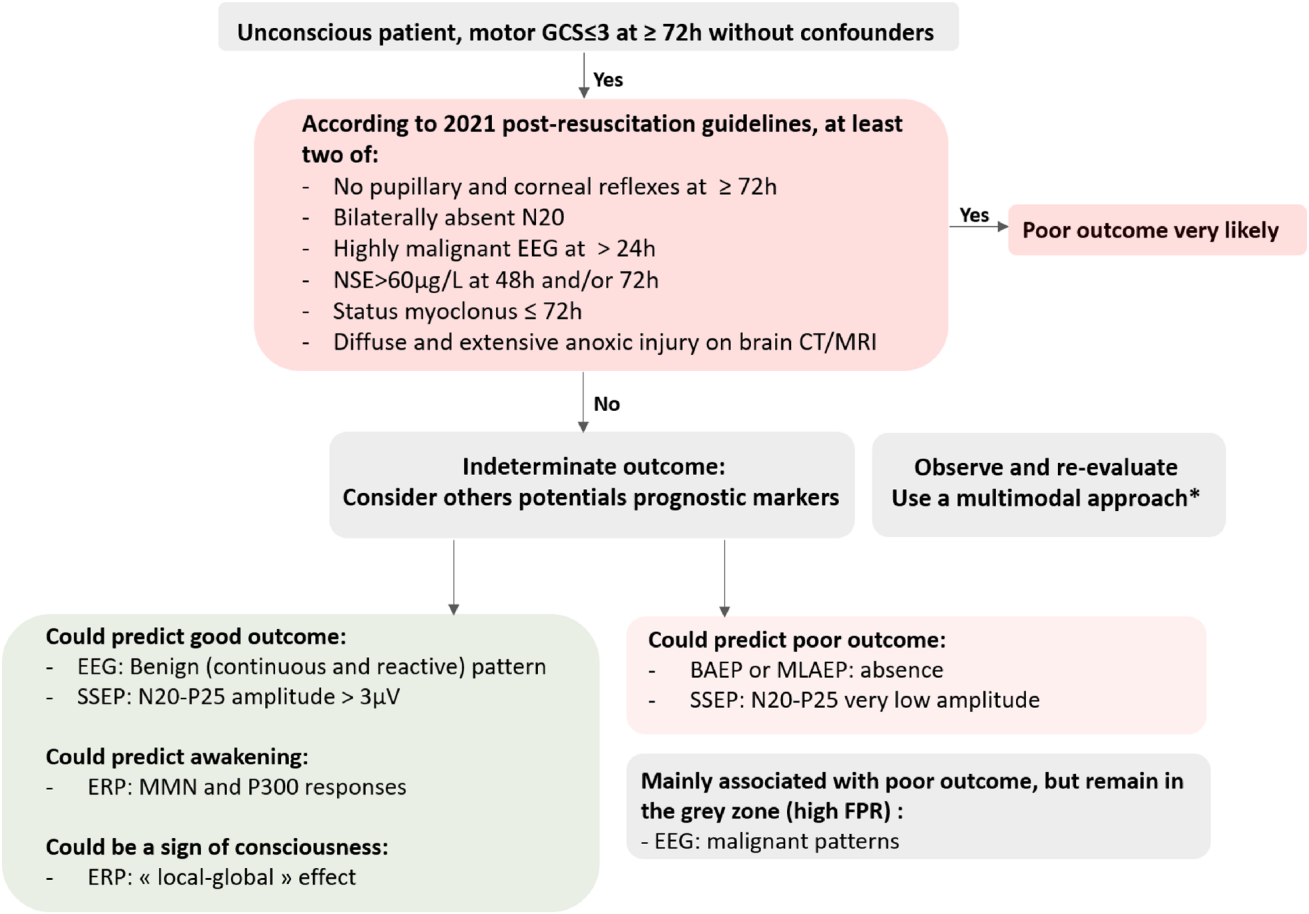
Algorithm of neuro-prognostication using neurophysiological markers, as a complement of the ERC/ESICM 2021 guidelines [4]. (*) We suggest to use a multimodal approach, including non-neurophysiological prognostic markers (biomarkers as NSE, brain imaging, pupillary and corneal reflexes and clinical status myoclonus). *BAEP* brainstem auditory evoked potential, *CT* computed tomography, *EEG* electroencephalogram, *ERP* event-related potential, *FPR* false positive rate, *GCS* Glasgow coma scale, *MLAEP* middle latency auditory evoked potential, *MRI* magnetic resonance imaging, *NSE* neuron specific enolase, *SSEP* somatosensory evoked potential

Evoked potentials (EPs) are neural responses time-locked to some stimulus. EPs differ from EEG signals as they are stimulus induced and represent the summated activities of large populations of neurons firing in synchrony, requiring the average of numerous stimulations. EP components are named according to their polarity (“N” for negative; “P” for positive) and their latency from the stimulation (in milliseconds). Different EPs (i.e., SSEPs, BAEPs, MLAEPs and long latency event-related potentials (ERPs) with mismatch negativity (MMN) and P300 responses) can be assessed at bedside in ICU patients, with different brain generators and prognostic values (Figs. 3, 4). A multimodal approach is recommended combining different EPs modalities to reduce the FPR and the risk of self-fulfilling prophecy risk [31, 106] (Fig. 5).

### Somato-sensory evoked potentials

Somato-sensory evoked potentials (SSEPs) allow evaluation of functional integrity of the somatosensory pathways [31, 107]. Median nerve SSEPs assess this functional integrity at different main levels: N9, generated by the proximal part of the median nerve; N13, generated by the posterior columns of the spinal cord; P14, generated at the cervico-medullar level; N20, generated by the primary somatosensory cortex. A five-channel device is recommended to record and analyze these distinct components (see Fig. 3 legend). A channel C’3–C’4 scalp electrodes were positioned 2 cm posterior to C3 and C4 (C3 corresponding to the left central and C4 to the right central electrodes, using the standard electrode position nomenclature [108]). This five-channel device is mandatory to differentiate the N20 cortical component from the N18 subcortical component. N20 is identified as the major negative peak (visible on the C’3–C’4 channel), while P25 is identified as the major positive peak following N20 (Fig. 3).

In most studies assessing neuro-prognostication value after CA, SSEPs are mostly recorded in patients still comatose 72 h after ROSC and 48 h after sedation discontinuation. Intravenous sedative drugs have little impact on SSEPs, while hypothermia (< 33 °C) and low blood pressure may have a depressant effect. Although SSEPs can be recorded 24 h after CA, expert recommendations suggest waiting 48 h after CA and discontinuing sedation for at least 6 h [31, 109] (Table 2). Concerning recording parameters, upper limbs SSEPs are elicited by electric stimulation of the right and left median nerve using a bipolar surface electrode at the wrist (stimulus duration 0.2–0.3 ms; stimulus intensity adjusted until the obtention of visible thumb twitches or reliable N9 at Erb point; stimulus frequency 3–5 Hz; usually average of three sets of  > 200 responses). Subdermal needle electrodes are recommended in the ICU environment to improve the quality of the signal. Neuromuscular blockades have no deleterious impact on SSEPs and should be considered to improve the signal to noise ratio, as the noise level should not exceed 0.25 µV. Interpretation of SSEP should be performed with a sensitivity of at least 1 µV/cm. Peripheral nerve lesions can lead to the absence of N9 and spinal cord injuries to the absence of N13 [107]. Upper limbs’ SSEPs thus need a documentation of N9 and N13 to eventually document a reliable bilateral N20 abolition. Inter observer variability seems less important for SSEPs than for EEG except in case of high noise level [110, 111]. N20 amplitude may be important for prognostication (see below), and can be measured as the N20 peak versus baseline or versus P25.

Concerning prognostic value, bilateral absence of N20 is recognized as the most robust marker of poor outcome (FPR 0%, CI95% (0.001–0.047)) [41, 112, 113] including in a population, where WLST was not performed [65]. Bilateral N20 absence probably reflects the severity of HIBI, as all patients with a bilateral N20 absence presented cortical and thalamic injuries [114]. This tool is thus already considered as one of the six prognostic markers of ERC/ESICM algorithm [4, 115].

By contrast, sensitivity of bilaterally absent N20 remains relatively low, around 30% [41, 116]; Moreover, presence of bilateral N20 is not predictive of good neurological outcome (PPV around 50%). To improve the prognostic value of SSEP**,** recent studies assessed the N20 and P25 amplitudes among patients with a bilateral presence of N20. For poor outcome prediction, a low voltage amplitude (between 0.40 and 1 µV according to studies [65, 70, 86, 117–120]) improved sensitivity from 30% to 50% compared to bilateral N20 absence, with a high specificity (93–100%). Conversely, a high N20–P25 amplitude (> 2.30 to 4 µV) [26, 117–121] predicts a favorable outcome with a high specificity (85–96%) but a moderate sensitivity (30–61%), although one study found no association with outcome [70]. Standardization of the method (i.e., SSEP N20-baseline or N20–P25 peak-to-peak measure) is needed to consider amplitude as a prognostic marker. A recent study highlighted that N20–P25 presented a higher prognostic value (AUC = 0.85) compared to N20-baseline (AUC = 0.70) [119]. Thus, N20 amplitude could be assessed as a continuum rather than a categorical variable, the underlying concept being that the amplitude of SSEP components could be inversely related to the severity of neurological injury.

### Brainstem auditory evoked potentials

Brainstem auditory evoked potentials (BAEPs) are recorded in response to the listening of monoaural clicks, in the 10 ms following stimulus onset. Five waves are observed, coming from different generators. Main generators are the distal portion of the auditory nerve (wave “I”), the bulbo-mesencephalic junction (wave “III”) ipsilateral to the stimulation side, and the inferior colliculus (wave “V”) (Fig. 4). BAEPs are poorly affected by sedative drugs, and could be recorded with moderate doses. Transient use of neuromuscular blockades may be useful to limit artefacts [31] (Table 2). BAEP abolition is highly correlated with UFO with a high specificity but a low sensitivity [41, 122–125]. Conversely, BAEPs preservation is not predictive of good outcome [126]. Importantly, BAEPs assessment is essential to confirm the integrity of peripheral and brainstem auditory pathways, to secondarily record middle and late auditory evoked potentials.

### Middle latency auditory evoked potentials (MLAEPs)

Middle latency auditory evoked potentials (MLAEPs) are elicited by monoaural clicks and can be recorded simultaneously to BAEPs (Fig. 4). MLAEPs are attenuated by sedative drugs and should be performed 48 h after sedation weaning (Table 2). Responses are expected in the 100 ms following the stimulus onset and is composed of two waves: Na and Pa. Bilateral abolition of Na and Pa responses is associated with UFO with a high specificity (100%) but a low sensitivity (37%) [126]. By contrast, their preservation has no prognostic value although one study found a correlation between MLAEP and ROSC, survival and neurologic outcome [127].

### N100 responses

N100 response is an auditory event-related potential (ERP), which reflects the activation of primary auditory cortices. The absence of N100 is considered to be predictive of a UFO, their recording being also an indispensable pre-requisite to record MMN [31].

### Long latency event-related potentials (ERPs)

Long latency event-related potentials (ERPs), also called endogenous potentials, are supposed to reflect a cognitive attention task. Accordingly, ERPs may provide relevant markers of cognitive functions in unresponsive state patients, and thus detect patients who could be in a “recovery process”. As ERPs responses are highly sensitive to the arousal state but also to sedation, ERPs should be performed in case of persistent unresponsive state 48 h after sedation discontinuation (Table 2). Neuromuscular blockades are often useful to limit artefacts. Many auditory paradigms have been described to elicit long latency ERPs but only a few of them are used for neuro-prognostication (Fig. 4).

### Mismatch negativity (MMN)

Auditory MMN is elicited by an “*oddball*” paradigm, in which series of standard frequent tones and deviant infrequent tones are played binaurally, without any active participation asked of the patient (i.e., passive paradigm). Standard and deviant tones differ in one of their acoustic characteristics (intensity, frequency, or duration) and their probabilities of occurrence (standard: ± 86% of time; deviant: ± 14%). MMN is obtained by subtracting the ERP of deviant tones from the ERP of standard tones on midline electrodes (Fz, Cz, Pz), between 100 and 250 ms post-stimulation (Fig. 4). According to surface and intracranial EEG, magneto-encephalography (MEG) and fMRI studies, MMN responses involve two main intracranial processes, the first one in the bilateral supratemporal cortices and the second predominantly in the frontal areas [128–131]. This response is supposed to reflect an automatic and pre-attentive detection of auditory violations [128]. Despite this, attention is a necessary prerequisite for consciousness, but possible dissociation between attention and consciousness has been demonstrated [132, 133].

About prognostic value, Fischer et al. assessed 62 DoC patients with ERPs, in a median time of 8 days after CA. In this study, the presence of MMN was a predictive marker of awakening (defined as neither dead nor permanent VS), with a PPV of 100% (CI95% (78–99)) and a negative predictive value (NPV) of 84% (CI95% (71–93)) [109]. These results were confirmed by further studies, including studies performed during therapeutic hypothermia [134–138]. In one study, some patients demonstrated a preserved MMN in a very acute stage (< 24 h after CA during hypothermia) and successively lost this response in a second MMN recording (performed  > 24 h after CA, during normothermia) [134]. None of these patients regained consciousness. Finally, MMN predicts awakening but does not exclude mild to severe neurological disability, as this response could be observed in around 35% of MCS patients and has been sometimes described in VS patients [9]. MMN also predicts awakening with an heterogenous sensitivity (between 27% and 100% among studies [9, 139–141]) emphasizing the need for additional prognostic markers [9, 141]. Moreover, due to the *oddball* paradigm with a few number of averaged deviant stimuli, MMN responses are sometimes difficult to distinguish from background activities. Accordingly, limited inter-rater agreement has been demonstrated [142]. Some authors proposed automated procedures with statistical validations. However, these procedures displayed contradictory results and should only be used as complementary to the visual analysis [143].

### P300 responses evoked by subject’s own name

P300 response is a complex positive response recorded during the oddball paradigm 300 to 350 ms after stimulus, if the patient focuses attention on deviant stimuli [144, 145] (Fig. 4). This response is amplified if the deviant stimulus is relevant for the patient. Thus, some studies demonstrated the effectiveness of the subject own-name stimuli to elicit the P300 response [146, 147]. *Oddball* paradigms have been developed to record simultaneously MMN and P300, also using scarce subject’s own-name for P300 recording [138, 139]. This P300 is supposed to express a brain evaluation of novelty before behavioral reaction [148]. Intracranial EEG and fMRI paradigms identified a widespread network behind the P300 response, from the prefrontal cortex to the inferior parietal areas [149–151].

About prognostic value, a limited cohort study suggested that P300 predicts awakening (defined as neither dead nor permanent VS or MCS) with a PPV of 100% (CI95% (61–100)), and a NPV of 93% (CI95% (66–100)) [138]. It is of importance to underline that P300 has mainly been evaluated in cohorts of sub-acute or chronic DoC patients (in 3 months after CA), the prognostic value of these responses being poorly evaluated at the acute stage of DoC after CA. Accordingly, P300 is an interesting marker for awakening prediction but does not seem to rule out mild to severe cognitive disabilities. Finally, visual interpretation of the P300 response may be sometimes challenging. Recommendations have been made for future statistical validations to be added to clinical practice [31]. These different results about prognostic value of P300 need to be confirmed in larger prospective studies.

### Active counting task: the «local–global» auditory paradigm

The «local–global» effect described by Bekinschtein [140] and colleagues is an active counting task, where patients listen to an auditory paradigm including local deviations (i.e., inside a series of five brief tones: all identical or only the last one different) and global deviation (i.e., on a longer time scale, successive series constitute a global regularity, which is violated by the irruption of 20% of different series). Patients are asked to count these “global deviations” which elicit a spatially cerebral distributed response (called the “global effect”) considered as a reliable marker of consciousness. Absence of “global effect” does not exclude residual consciousness as in healthy subjects, global effect disappeared if subjects are distracted by a visual interference task [140]. The same authors confirmed the high PPV (close to 100%) of this “global effect” to probe consciousness, despite a low NPV [152]. However, Tzovara et al. identified this “global effect” in only 10/24 post-anoxic comatose patients (including 5 sedated patients) [153]. These significant discrepancies from previous studies could be explained firstly by the differences in the timing of DoC assessment (earlier than previous studies), second by the difference in acoustical characteristics of stimuli and third by the difference in EEG analysis and statistic variability [143, 154]. Further studies are mandatory to evaluate this prognostic marker.

To highlight and summarize the interest of these different tools in patients with “indeterminate neurological outcome” after CA, we propose an algorithm of neuro-prognostication involving these potentials neurophysiological prognostic markers, use as a complement of the ERC/ESICM 2021 guidelines (Fig. 5).

## Conclusion

In comatose cardiac arrest patients, recent advances in clinical research now allow a better use of neurophysiological tools, including increased discernment in a multimodal approach. Importantly, their availability currently remains the main limitation to their routine use in intensive care units. Finally, if their efficacy is firmly established for the prediction of an unfavorable neurological evolution, ongoing research will probably make it possible to use them to better predict a favorable evolution in the next future.

## Data Availability

We confirm that the figures are original and have not been published elsewhere.

## Abbreviations

ACNS: American clinical neurophysiology society
BAEP: Brainstem auditory evoked potentials
CA: Cardiac arrest
cEEG: Continuous EEG
CI: Confidence interval
CMD: Cognitive-motor dissociation
CMS: Cortically mediated state
CPC: Cerebral performance category
DoC: Disorders of consciousness
EEG: Electroencephalogram
EER-r: EEG reactivity
ERP: Event-related potentials
FPR: False positive rate
GCS: Glasgow coma scale
GOS-E: Glasgow outcome scale-Extended
HIBI: Hypoxic ischemic brain injury
ICU: Intensive-care-unit
IQR: Interquartile-range
MCS: Minimal consciousness state
MEG: Magneto-encephalography
MLAEP: Middle latency auditory evoked potentials
MMN: Mismatch negativity
mRS: Modified Rankin Scale
NPV: Negative Predictive Value
NSE: Neuron Specific Enolase
PPV: Positive Predictive Value
qEEG: Quantitative EEG
RASS: Richmond–Agitation–Sedation-Scale
rCRI: Revised cerebral recovery index
ROSC: Return of Spontaneous Circulation
SD: Standard Deviation
SE: Status epilepticus
Se: Sensitivity
SIRPIDS: Stimulus-induced rhythmic or periodic discharges
Sp: Specificity
SSEP: Somatosensory Evoked Potentials
TTM: Targeted Temperature Management
UFO: Unfavorable outcome
VS: Vegetative state
WLST: Withdrawal-of-Life-Sustaining-Treatments
µV: MicroVolt
